# Concept libraries for automatic electronic health record based phenotyping: A review

**DOI:** 10.23889/ijpds.v6i1.1362

**Published:** 2021-06-16

**Authors:** Zahra A Almowil, Shang-Ming Zhou, Sinead Brophy

**Affiliations:** Swansea University Medical School, Wales SA2 8PP; Centre for Health Technology, Faculty of Health, University of Plymouth, Plymouth, PL4 8AA, UK

**Keywords:** linked Electronic health records, phenotype algorithms, concept libraries, review

## Abstract

**Introduction:**

Electronic health records (EHR) are linked together to examine disease history and to undertake research into the causes and outcomes of disease. However, the process of constructing algorithms for phenotyping (e.g., identifying disease characteristics) or health characteristics (e.g., smoker) is very time consuming and resource costly. In addition, results can vary greatly between researchers. Reusing or building on algorithms that others have created is a compelling solution to these problems. However, sharing algorithms is not a common practice and many published studies do not detail the clinical code lists used by the researchers in the disease/characteristic definition. To address these challenges, a number of centres across the world have developed health data portals which contain concept libraries (e.g., algorithms for defining concepts such as disease and characteristics) in order to facilitate disease phenotyping and health studies.

**Objectives:**

This study aims to review the literature of existing concept libraries, examine their utilities, identify the current gaps, and suggest future developments.

**Methods:**

The five-stage framework of Arksey and O'Malley was used for the literature search. This approach included defining the research questions, identifying relevant studies through literature review, selecting eligible studies, charting and extracting data, and summarising and reporting the findings.

**Results:**

This review identified seven publicly accessible Electronic Health data concept libraries which were developed in different countries including UK, USA, and Canada. The concept libraries (n = 7) investigated were either general libraries that hold phenotypes of multiple specialties (n = 4) or specialized libraries that manage only certain specialities such as rare diseases (n = 3). There were some clear differences between the general libraries such as archiving data from different electronic sources, and using a range of different types of coding systems. However, they share some clear similarities such as enabling users to upload their own code lists, and allowing users to use/download the publicly accessible code. In addition, there were some differences between the specialized libraries such as difference in ability to search, and if it was possible to use different searching queries such as simple or complex searches. Conversely, there were some similarities between the specialized libraries such as enabling users to upload their own concepts into the libraries and to show where they were published, which facilitates assessing the validity of the concepts. All the specialized libraries aimed to encourage the reuse of research methods such as lists of clinical code and/or metadata.

**Conclusion:**

The seven libraries identified have been developed independently and appear to replicate similar concepts but in different ways. Collaboration between similar libraries would greatly facilitate the use of these libraries for the user. The process of building code lists takes time and effort. Access to existing code lists increases consistency and accuracy of definitions across studies. Concept library developers should collaborate with each other to raise awareness of their existence and of their various functions, which could increase users’ contributions to those libraries and promote their wide-ranging adoption.

## Introduction

Electronic health records (EHR) have been adopted across the UK. For example, in terms of primary care in the UK there are the following four databases: 1) CPRD (Clinical Practice Research Data Link); 2) THIN (The Health Improvement Network); 3) QResearch and 4) SAIL (Secured Anonymised Information Linkage) in Wales [2]. In addition, secondary care data such as the hospital admission system (HES – England, PEDW – Wales, SMR -Scotland), are linked to primary care records [1 - 4]. Such linked information creates the opportunity to undertake research into the causes and outcomes and pathway of disease. However, using linked routine data requires some specialist skills, for example, using the data requires: 1) identifying conditions of interest from diagnosis, treatments, and procedures, and 2) creating phenotype algorithms (such as diagnosis of rheumatoid arthritis and medication for rheumatoid arthritis) and developing specific inclusion and exclusion criteria [5].

The construction of phenotype algorithms enables repeatable research and ensures that different researchers are using the same standards to identify patients [6]. However, the process of constructing phenotype algorithms is very time consuming and resource costly [7], and so reusing previously created phenotype algorithms to conduct repeatable research becomes a compelling solution. However, it is not common for researchers to share their clinical code lists in their published studies [8]. Therefore, it is difficult to make comparisons between studies as different studies often have different definitions of the same condition [4].

Although clinical code lists were published along with some EHR based studies, researchers often find it difficult to extract the relevant parts from lists for other research studies. Consequently, it is difficult to evaluate the transparency of EHR based research [9]. Even though researchers request better transparency in publishing clinical code lists [10, 11], currently journals and funding parties do not make it mandatory to publish code lists [9].

To address these challenges and ensure scientific transparency, data linkage centres have developed concept libraries for disease phenotyping, working as platforms to enable storing, managing, and sharing of phenotypes (Diagnoses, Symptoms, Medications and Procedures) by multiple researchers. For example, ClinicalCodes.org and CALIBER in the UK, and The Concept Dictionary and Glossary in Canada [9, 12, 13]. In the literature, concept libraries for disease phenotyping have different names and various definitions. We aim to review the literature of existing concept libraries to examine how they are used, identify the current gaps and future development. By evaluating the existing concept libraries and scoping what is missing in the current environment, this study could facilitate the development and improvement of concept libraries.

## Methods

The five-stage framework of Arksey and O'Malley was used for the literature search [14]. This approach included defining the research questions, identifying relevant studies through literature review, selecting qualified studies, charting and collecting data, and summarising and reporting the findings. 

### Data Sources

This stage involved identifying the research questions, which provided the roadmap for subsequent stages. The questions to be addressed were:

- What concept libraries already exist?
- What are their features? Are there similarities or differences among them?

### Identification of relevant studies

This stage involved identifying the relevant studies and developing a decision plan for where to search, which terms to use, which sources are to be searched, time span, and language. Searching was limited to peer reviewed manuscripts which were written in the English language and were published from 2010 to 2019. Five databases were searched including Medline, CINAHL, LISTA, Google Scholar, and Web of Science using the following sets of key words:

1. "electronic health record*" or "electronic medical record*" or "computerized health record*" or "computerized medical record*" or EHR or EMR
2. portal* or platform* or repositor* or library* or dictionary*
3. phenotyp* or e-phenotyp* or phenomic* OR "clinical code list*" or "clinical code*" or "clinical concept*" OR "clinical code set*" or "clinical value set*"
4. The sets of key words have been altered to be used in Google Scholar as recommended by this database as follows: ("electronic health record*" or "electronic medical record*" or EHR or EMR) AND (phenotyp*) AND (portal* or platform* or repository* for library* or dictionary*)

### Selecting of eligible studies

**Figure 1: Overview of the steps taken in the priority-setting process.**
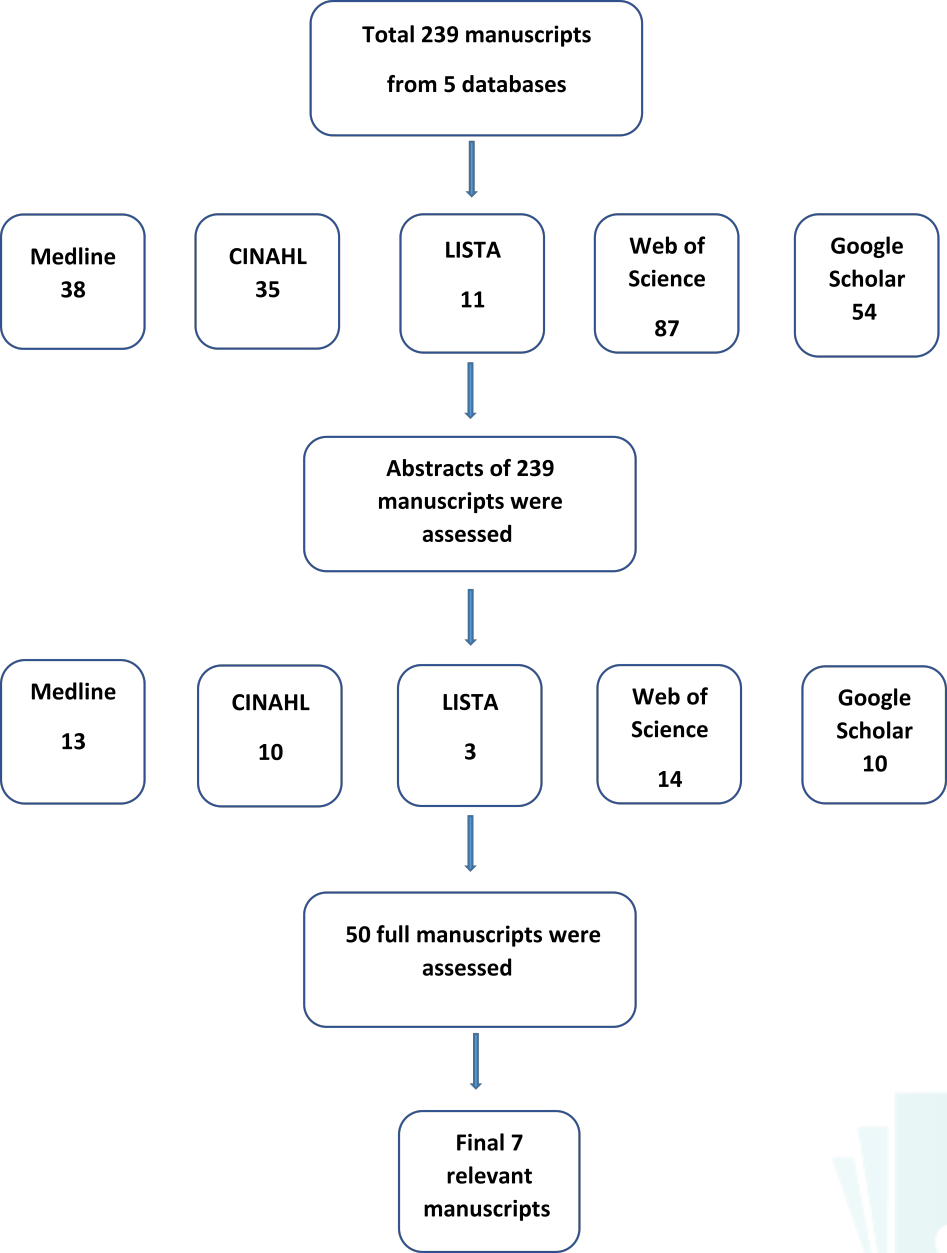


The first author reviewed all the abstracts of the identified manuscripts (n=239) based on their relevance to the research questions. Those with relevant abstracts were taken forward to full assessment (n=50). Out of the fifty fully assessed manuscripts, only seven were selected as they matched the planned inclusion and exclusion criteria. The inclusion criteria for the selection process were to include manuscripts about public concept libraries for electronic linked health data-based phenotyping, and their different definitions, types, and functions, such as allowing users to share, reuse, and verify research methods (e.g., code lists, algorithms, and metadata). Manuscripts related to electronic health record phenotyping authoring tools are excluded. Figure. 1 depicts more information about the selection process of the related studies, and Table. 1 presents an overview of the seven concept libraries including their definitions/purposes, electronic data sources, coding systems, and examples of phenotype definitions in the seven public concept libraries.

**Table 1: An overview of the seven concept libraries:** 

Concept Libraries	Definitions/Purposes	Developers/Leaders	References of the Manuscripts/URL Access of the Concept Libraries	Electronic data sources/Coding systems	Examples of phenotypes
ClinicalCodes.org	An online repository that contains a set of published studies. For each study a code list or a group of code lists has been uploaded on the ClinicalCodes.org site. Code lists are publicly accessible to improve validity and reproducibility of electronic medical record studies.	The University of Manchester. Institute of Population Health, UK	9. Spring ate DA, Ketopantoic E, Ashcroft DM, Olier I, Parisi R, Chamapiwa E, et al. ClinicalCodes: An online clinical codes repository to improve the validity and reproducibility of research using electronic medical records. 2014; 9(6):6–11. https://clinicalcodes.rss.mhs.man.ac.uk/	Primary and secondary care using Read, OXMIS, SNOMED, CPRD, product/medical code, BNF code, ICD-9, ICD-10	Research article: Are symptoms of insomnia in primary care associated with subsequent onset of dementia? A matched retrospective case-control study, Link to the shared phenotypic descriptions at: https://clinicalcodes.rss.mhs.man.ac.uk/medcodes/article/78/
CALIBER data portal	An open online repository of phenotyping algorithms that contains all definitions of research variables using CALIBER data sources in order to encourage research and promote transparency.	Led from the University College London (UCL) Institute of Health Informatics, UK	21. Dewaxes S, Gonzalez-Inquired A, Direk K, Fitzpatrick NK, Fatemifar G, Banerjee A, et al. UK phenomics platform for developing and validating electronic health record phenotypes: CALIBER. J Am Med Inform Assoc. 2019; 26(12):1545–59. https://www.caliberresearch.org/portal/phenotypes	Primary care, hospital records, social deprivation information, cause-specific mortality data. Using Read codes (a subset of SNOMED-CT), ICD-9, ICD-10, OPCS-4 (analogous to Current Procedural Terminology terms) and Gemscript.	Abdominal Hernia: “At the specified date, a patient is defined as having had Abdominal Hernia If they meet the criteria for any of the following on or before the specified date. The earliest date on which the individual meets any of the following criteria on or before the specified date is defined as the first event date: Primary care 1. Abdominal Hernia diagnosis or history of diagnosis or procedure during a consultation OR Secondary care 1. ALL diagnoses of Abdominal Hernia or history of diagnosis during a hospitalization OR Secondary care (OPCS4) 1. ALL procedures for Abdominal Hernia during a hospitalization” Link to the shared phenotypic descriptions at: https://www.caliberresearch.org/portal/phenotypes/chronological-map
The MCHP Concept Dictionary and Glossary	The Concept Dictionary includes comprehensive operational definitions and programming code for measurements used in MCHP research including a description of the problem(s) involved, methods used, and programming tips/ cautions, and the Glossary records terms that are widely used in research based on population. The Concept Dictionary was developed to help researchers use reliable, validated algorithms to perform methodologically comprehensive research.	The Manitoba Centre for Health Policy (MCHP), Canada	13. Soapy T. Manitoba Centre for Health Policy Data Repository. In: Michalos AC (eds) Encyclopaedia of Quality of Life and Well-Being Research. Springer, Dordrecht; 2014. http://umanitoba.ca/faculties/health_sciences/medicine/units/chs/departmental_units/mchp/resources/concept_dictionary.html	- The MCHP databases: Health, Education, Social, Justice, Registries, Support Files. - Operational definitions and SAS program code for variables or measures developed from administrative data. - The International Classification of Disease (ICD) diagnoses or ICD / CCI (Canadian Classification of Health Interventions) procedure / intervention	Manitoba Asthma Algorithms The following is an example of asthma algorithm developed by a research project. “Raymond et al. (2011) use a broader scope in their definition for asthma, defining it as one physician claim OR one hospital claim with a corresponding diagnosis of: ICD-9-CM: 464, 466, 490, 491, 493 or ICD-10-CA: J04, J05, J20, J21, J40, J41, J42, J45, J441, J448 OR one prescription for an asthma medication in a three-year period. “ Link to the shared phenotypic descriptions at: http://mchp-appserv.cpe.umanitoba.ca/viewConcept.php?conceptID=1305#a_references
Phenotype knowledgebase (PheKB)	An online environment supporting the workflow of building, sharing, and validating electronic phenotype algorithms. The PheKB was designed to facilitate the transportability of algorithms into various research applications across different organizations, health care systems, and repositories of clinical data.	Led by Vanderbilt University, (the eMERGE Network Coordinating Center), USA	19. Kirby JC, Speltz P, Rasmussen L V., Basford M, Gottesman O, Peissig PL, et al. PheKB: A catalogue and workflow for creating electronic phenotype algorithms for transportability. J Am Med Informatics Assoc. 2016; 23(6):1046–52. https://phekb.org/	Clinical and genomic data from electronic health records. HCPT Codes, ICD 10 Codes, ICD 9 Codes, Laboratories, Medications, Natural Language Processing	Urinary Incontinence The cohort is defined with the following criteria: a. EHR of all male patients of 35 years of age or more, AND b. For which there is an ICD-9-CM / ICD-10-CM diagnosis of prostate cancer, AND c. For which there are at least two encounters before first treatment, AND d. For which there is at least one clinical note before first treatment, AND e. For which there is either prostatectomy surgery or radiation procedure performed as identified by CPT codes. Link to the shared phenotypic descriptions at: https://phekb.org/phenotype/1404

2. Specialized libraries					

Genome-Phenome Analysis Platform (GPAP)	An online data platform, where data from sequencing experiments contributed by collaborating research projects is processed using a standard pipeline and made accessible to registered users for online analysis through a user-friendly interface.	It was developed by RD-Connect and Led by Aix-Marseille University Medical School (AMU), France	27. Thompson R, Johnston L, Taruscio D, Monaco L, Béroud C, Gut IG, et al. RD-Connect: An integrated platform connecting databases, registries, biobanks and clinical bioinformatics for rare disease research. J Gen Intern Med. 2014; 29(SUPPL. 3):780–7. https://dx.doi.org/10.1007%2Fs11606-014-2908-8	Genomic and clinical data from RD-Connect’s partners rare disease-based research projects. The PhenoTips database stores phenotypic profiles for individual cases coded by human phenotype ontology (HPO). A directory of biobanks and patient registries and a bio sample catalogue.	Case 1: description RD-Connect identifier: Case1C Gender: Male, Age: 5 years, Referral: Congenital myasthenic syndrome, Onset: Congenital, Global pace of progression: Progressive (slow), Main clinical features: Neonatal hypotonia, Distal arthrogryposis, Inability to walk, Recurrent lower respiratory tract infections. Link to the shared phenotypic descriptions at: https://playground.rd-connect.eu/
The PhenoScanner V2	A database that contains publicly existing results of large-scale genomic association studies. It was developed to facilitate the cross-referencing of genetic variants with a wide variety of phenotypes for better comprehension of biology and pathways of disease.	The Cardiovascular Epidemiology Unit, University of Cambridge, UK	24. Kamat MA, Blackshaw JA, Young R, Surendran P, Burgess S, Danesh J, et al. PhenoScanner V2: an expanded tool for searching human genotype-phenotype associations. Bioinformatics. 2019; 35(22):4851–3. http://www.phenoscanner.medschl.cam.ac.uk/	137 genotype–phenotype association datasets, including results for anthropometric traits, blood pressure, lipids, cardiometabolic diseases, renal function measures, glycemic traits, inflammatory diseases, psychiatric diseases and smoking phenotypes. It also includes the NHGRI-EBI GWAS catalog, and dbGaP catalogues of associations.	Trait: Crohn's disease “A gastrointestinal disorder characterized by chronic inflammation involving all layers of the intestinal wall, noncaseating granulomas affecting the intestinal wall and regional lymph nodes, and transmural fibrosis. Crohn disease most commonly involves the terminal ileum; the colon is the second most common site of involvement. A chronic transmural inflammation that may involve any part of the DIGESTIVE TRACT from MOUTH to ANUS, mostly found in the ILEUM, the CECUM, and the COLON. In Crohn disease, the inflammation, extending through the intestinal wall from the MUCOSA to the serosa, is characteristically asymmetric and segmental. Epithelioid GRANULOMAS may be seen in some patients.” Link to the shared phenotypic descriptions at: https://www.ebi.ac.uk/gwas/efotraits/EFO_0000384
Genotypes and Phenotypes Database (dbGap)	A National Institute of Health-sponsored repository tasked with archiving, curating and distributing information provided by studies examining genotype and phenotype interactions. It was developed with standardized identifiers that allows published studies to address or cite the primary data in a clear and uniform way.	National Centre for Biotechnology Information, USA	15. Tryka KA, Hao L, Sturcke A, Jin Y, Wang ZY, Ziyabari L, et al. NCBI’s database of genotypes and phenotypes: DbGaP. Nucleic Acids Res. 2014; 42(D1):975–9. https://www.ncbi.nlm.nih.gov/gap/	Genetic and phenotypic databases sponsored by NIH and other agencies around the world including: Genotype, phenotype, exposure, expression array, epigenomic and pedigree data from genome-wide association studies (GWAS), sequencing studies and other large-scale genomic studies.	“Autism_Genome_Project_Subject_Phenotypes: The subject phenotype table includes data collected on sociodemography (n=2 variables; sex and European ancestry) and psychological and psychiatric observations (n=8 variables; spectrum and strict definition of autism, whether the subject is non-verbal and/or verbal, has low or high IQ, and the age of their first word and phrase). This table now also includes the stage of the study in which the individual was present and whether the individual is a member of a multiplex or simplex family.” Link to the shared phenotypic descriptions at: https://www.ncbi.nlm.nih.gov/projects/gap/cgi-bin/variable.cgi?study_id=phs000267.v5.p2&phv=161303&phd=3659&pha=3690&pht=2305&phvf=&phdf=&phaf=&phtf=&dssp=1&consent=&temp=1

### Extraction, charting, and synthesis of data

Data was extracted from the seven related manuscripts using a data-charting form. A narrative review method was used to extract data about the investigated seven public concept libraries for electronic linked health data-based phenotyping including their names, types, and characteristics such as enabling users to share, validate, and reuse of research methods such as algorithms. 

### Collecting, summarising and reporting the findings

A thematic construction was used to provide an overview of the breadth of the literature, and then a thematic analysis was used to generate the results. The different types and the characteristics of the seven public concept libraries were summarised. The types of electronic data sources used in each library (e.g., primary or secondary care or genetic data) and the used coding system (e.g., Read, OXMIS, ICD-9, and ICD-10) were all reported.

## Results

### Identified public concept libraries from the literature

There were seven public concept libraries from the literature developed by different countries including UK, USA, and Canada. These libraries were the ClinicalCodes.org [9], the Genotypes and Phenotypes Database (dbGaP) [15], Phenotype knowledgebase (PheKB) [18], the Manitoba Centre for Health Policy (MCHP) Concept Dictionary and Glossary [20], Clinical Disease Research using Linked Bespoke Studies and Electronic Health Records (CALIBER) [21], the PhenoScanner V2[22], and The Genome-Phenome Analysis Platform (GPAP) [24]. Four of the libraries were general libraries and held concepts and phenotypes on multiple specialities ranging from specific conditions (such as codes to identify lupus) to general demographic concepts (such as smoking status). Three of the libraries were specialised libraries and only give concepts on certain defined areas such as rare diseases. However, in common across all the libraries was that they allowed users to share by uploading their own concepts, to examine validity of concepts by showing where they were published, and all had the aim of facilitating reuse of research methods such as clinical code lists or metadata.

There were some clear differences between the general libraries such as archiving data from different electronic sources (e. g. primary care, secondary, social deprivation information, cause-specific mortality data, health, education, justice, and registries); using various types of coding systems (e.g. SNOMED, BNF, READ, ICD9 /10, and CCI (Canadian Classification of Health Interventions) [9] [18] [20] [21]; having different policies that govern accessing the underlying data sources (e.g. a researcher has to complete the Data Access Process (DAP) of the Manitoba Centre for Health Policy (MCHP) to access the data and conduct research by using the Manitoba Population Research Data Repository) [20]; and allowing different searching queries such as simple or more advanced searches (e.g. CALIBERcodelists package enables users to search for code lists by synonym or code stub, and combine search terms using Boolean operators) [29].

However, they share some clear similarities such as having similar purposes (e.g. helping researchers to perform comprehensive research, promoting transparency in sharing research methods, and improving reproducibility of studies; enabling users to upload their own code lists and other important documents (e.g. users of PheKB can upload related documents and their phenotypes along with multidimensional metadata labels, documents including detailed descriptions of the computable algorithms such as types of used data, logic of execution, definitions of data, and flow charts) [18]; and allowing users to use/download the publicly accessible code lists (e.g. users of ClinicalCodes.org can download a file containing all codes associated with a study as csv files) [9].

There were some differences between the specialized libraries such as using data that were generated by various electronic databases (e.g. the PhenoTips database, database of biobank and patient registries, the NHGRI-EBI GWAS catalogue, and genetic and phenotypic databases sponsored by NIH and other agencies around the world) [15] [22] [25]; allowing diverse searching strategies (e.g. registered users of GPAP may select one or more individuals such as trios or other family relationships to explore and then filter and refine the outcomes by inheritance mode, population frequencies, tools for in silico pathogenicity prediction, gene lists and Linvar, HPO and OMIM codes)[30]; and enabling different searching queries such as simple or complex searches (e.g. all publicly released dbGaP studies can be queried by users. Queries can be very simple, just a keyword of interest (‘cancer’) or complex, making use of search fields and Boolean operators (‘cholesterol[variable] AND phs000001’) [15].

Conversely, there were some similarities between the specialised libraries such as enabling users to share by uploading their own concepts in the libraries to analyse the validity of concepts by showing where they were published (e.g. the GPAP enables clinicians and researchers who upload patient datasets to analyse their own data [30], and NIH-funded researchers can share their produced data, in the dB Gap) [15]; all aimed to encourage the reuse of research methods such as lists of clinical code or metadata (e.g. registered users of GPAP are allowed to access and search data sets provided by other researchers on similar patients[30], and users of the PhenoScanner V2 can use the archived findings from large-scale genetic association studies which are publicly accessible) [22]; and allowing access to some datasets through specific established control access (e.g. individual level data is accessible in the dbGap to scientists around the world through controlled application of access) [15]. Information about all the seven concept libraries such as their access URL and references of the seven manuscripts are presented in Table 1. 

### An overview of some the seven public concept libraries’ features

1. **Names and Definitions:**
Each of the investigated concept libraries has a specific name and a unique definition (Table 2 ). For example, CALIBER is defined as "a unique research platform consisting of ‘research ready’ variables extracted from linked electronic health records (EHR) from primary care, coded hospital records, social deprivation information and cause-specific mortality data in England" ( https://www.ucl.ac.uk/health-informatics/caliber ). Whereas, the Database of Genotypes and Phenotypes (dbGap) is defined as “a National Institutes of Health-sponsored repository charged to archive, curate and distribute information produced by studies investigating the interaction of genotype and phenotype" [15].
2. **Types**
***2.1 The general concept libraries:***
**The ClinicalCodes online repository**
The ClinicalCodes repository contains a selection of published studies that have been uploaded to the ClinicalCodes.org site along with a code list or a series of code lists. A code name, coding system (Read, OXMIS, SNOMED, CPRD product / medical code, BNF code, ICD-9, ICD-10), definition and type of entity (diagnostic, drug, examination, clinical sign, administrative, demographic, observational, immunization) are assigned to all individual clinical codes. Metadata and links to studies code lists are accessible as research objects that could be shared in machine-readable form throughout platforms. A research object file of JavaScript Object Notation (JSON) is available for each study that contains metadata (title, author, abstract, reference, link, DOI), commentary on the study level, commentary on the code list level and links to the individual files of the code list. Such object research files are directly accessible when inserting a '/ro' to the URI for a study e.g., ( www.clinicalcodes.org/medcodes/article/5/ro ) [9]. The developers of the ClinicalCodes repository have created an open-source R package (rClinicalCodes) to automate the downloading and importing lists of clinical code and metadata through the research object file from the repository website: ( https://cran.r-project.org/web/packages/rClinicalCodes/index.html . The developers of the ClinicalCodes repository will implement in the future: 1) Searching and downloading of codes by disease group, keyword and/or code group. 2) Methods for downloading code-lists and article metadata in machine readable form. 3) An API for downloading code-lists programmatically [9].
**The Clinical Research using Linked Bespoke Studies and Electronic Health Records (CALIBER)**
  CALIBER has developed the CALIBERcodelists package to manage ICD-10, Read and OPCS coding lists to identify medical conditions for research using CALIBER or other UK electronic health record databases. The package is written in R language, but many of the functions are accessible through an interactive menu and do not require any experience with R. The package has many features: 1) provides a standardized approach to identify codes of interest including Read, ICD-10 and OPCS through searching for term text or codes, 2) enables displaying of code lists on a spreadsheet and removing individual terms or modifying their categories, 3) downloads code lists in a variety of formats, and uploads them in default file format, 4) allows comparing of one code list to another, or combine two code lists together, 5) enables converting of code lists across dictionaries using the NHS mapping between the terminologies of Read/OPCS and Read/ICD-10, 6) processes a document which contains code to produce a code list and a descriptive text, and produces a comprehensive HTML document and a standardised format code list [27].
**The Manitoba Centre for Health Policy (MCHP) Concept Dictionary and Glossary**
  MCHP has built a range of web-based tools that record the historical usage of the repository-saved information such as the MCHP Concept Dictionary and Glossary [20]. Although there are short definitions for widely used terminology in the glossary, the Concept Dictionary includes comprehensive operational definitions and programming code for measurements used in MCHP research. A coherent documentation approach is used to describe the research methodologies. They can be presented either as best practices, or as different versions of historical overviews. Enhancements substitute older versions with a best-practice approach which requires authoritative approval of what is "the best", and the historical overview records all published methods for making options accessible to the user and information about the methodologies used in previous studies [26].
**The Phenotype Knowledgebase (PheKB)**
  The Phenotype Knowledgebase (PheKB accessible at http://phekb.org) was created within the eMERGE Network as a workflow management system and learning centre to support computable algorithm creation, validation, and sharing. It enables the transportability of algorithms across various research applications, multiple organizations, health care systems, and clinical data repositories through feedback processes and standardised implementation performance measures. PheKB contains built-in tools designed specifically to improve sharing of knowledge across sites, for example, the Data Dictionary / Data Validation Tool and the data management function. The Data Dictionary / Data Validation Tool is a registered user embedded resource that validates definitions of covariate data and related data, promotes data standardization, and early-stage quality assurance to exchange data for study sets efficiently. It is used for identifying errors and warnings in data dictionaries and data files related to a given phenotype through a custom Drupal module. It can show errors and warnings regarding the structure and content of the files as files are uploaded, while the data management function provides tracking tools for users to easily determine what data has been shared, what algorithm it is linked to, and by whom it was shared [18].
***2.2 The specialized concept libraries:***
**The Genome-phenome analysis platform (GPAP)**
  The RD-Connect built an integrated Genome-phenome analysis platform (GPAP), which is a user-friendly tool for diagnosing and discovering genes [30]. It links anonymised omics and clinical data with tools and services to examine these data online. The main portal provides links to the genomics analysis interface and the Phenotypes database that store ontology of phenotypic profiles coded for individual cases by human phenotype (HPO). GPAP also includes a database of biobanks and patient registries, and a catalo of bio samples that allows information of individual samples housed in participating biobanks to be drilled down [17]. For example, a researcher may select one or more individuals (e.g., trios or other family relationships) to explore and then filter and refine the outcomes by inheritance mode, population frequencies, tools for in silico pathogenicity prediction, gene lists and Linvar, HPO and OMIM codes [25] [30].
**The Pentosane V2**
  The developers of PhenoScanner V1 have collected more than 5,000 genotype-phenotype association datasets to create version 2 of the catalogue (PhenoScanner V2). PhenoScanner V2 has an API that contains an R package and a Python command line tool associated with it, which enables users to search for PhenoScanner V2 genotype-phenotype associations within R or from a terminal. All results, irrespective of P-value, can be presented when querying genetic variants, allowing the user to find indication against phenotype associations. PhenoScanner V2 has new features to facilitate improved 'phenome scans' including: 1) an expanded database of human genotype-phenotype associations divided into phenotype classes (diseases and traits, gene expression, proteins, metabolites and epigenetics), 2) new search selections such as gene, genomic region and queries based on phenotypes 3) linkage disequilibrium (LD) information for the five super-ancestries in 1000 Genomes 4) variant annotation and trait ontology mappings 4) annotation variations and ontology mappings of traits, and 5) a new Platform and API [22].
**The database of Genotypes and Phenotypes (dbGaP)**
The Genotypes and Phenotypes database (dbGaP) enables licensed users to identify and display different regions of the human genome, such as all the Allele frequencies and subgroups of individual-level genotype as well as sequence data, which are stored in that region in dbGaP, without accessing data sets of interest and performing multiple analyses [23]. The browser uses the standard graphical interface built for data from the 1000 Genomes Project and dbGaP by the National Centre for Biotechnology Information (NCBI), which incorporates sequence viewer track views with genotype tables and a novel sample / subject data selector showing core sample phenotype data [15]. The webpage of the browser includes a selection of 'widgets' pages showing data from the dbGaP view-only data project, which is data from the collection of dbGaP general research usage (GRU). The widgets work in such a way that one widget operation causes updating of other widgets on the page. See online browser documentation ( https:/www.ncbi.nlm.nih.gov/gap/ddb/help/ ) and The NCBI YouTube channel ( https:/www.youtube.com/user/NCBINLM ) for additional widget information.
3. **Characteristics**
  Some of the characteristics of the seven concept libraries are presented in Table 2.
**3.1 Sharing of Concepts**
  All of the identified concept libraries allow researchers to share some or all of their research methods such as clinical codes list, metadata, and algorithms. For example, users of the ClinicalCodes.Org are able to upload the code list and metadata for specific codes, and add comments at the code list, or study level. However, an account should be created first [9]. According to the developers of ClinicalCodes.Org, “To date: 93375 clinical codes have been deposited over 521 code lists” [19]. Similarly, Phenotype knowledgebase (PheKB) enables researchers to upload related documents and their phenotypes along with multidimensional metadata labels including the methods used in the phenotype standards such as International Classification of Disease (ICD) codes, medications, and natural language processing (NLP). Researchers also can upload documents that include detailed descriptions of the computable algorithms, such as types of data used, logic of execution, definitions of data, and flow charts [18].
  Builders of the Concept Dictionary and Glossary at the Manitoba Centre for Health Policy (MCHP) encourage researchers to share their discoveries, such as creating new concepts or updating existing concepts to grow and improve the value of this publicly accessible resource. Their Concept Dictionary describes more than 300 research concepts developed at MCHP for the analysis of data contained in the data warehouse hosted at MCHP [20]. Also, the developers of the CALIBER platform promote collaborative research. They compiled more than 90,000 terms from five standardised clinical terminologies to construct 51 validated phenotyping algorithms (35 diseases or syndromes, 10 biomarkers, 6 risk factors for lifestyles) [21]. All data sources are made accessible to researchers and can be accessed in a secure data-safe haven environment located at UCL IHI / Farr Institute, London or could be accessed remotely. Due to the varied clinical backgrounds of the datasets, they offer training on data sources, coding, consistency and management with the CALIBER team [16].
The Phenoscanner V2 database includes more than 5000 genetic association datasets from publicly accessible datasets of complete summary of associations findings collected by the NHGRI-EBI ( https:// www.ebi.ac.uk/gwas/downloads/summary-statistics ) and NHLBI ( https://grasp.nhlbi.nih.gov/FullResults.aspx ), and recent literature reviews and GWAS omics datasets [22]. Also, the Genotypes and Phenotypes Database (dbGaP) allows sharing of information obtained from studies examining genotype and phenotype interactions [15]. These studies include research of genome, medical sequencing, molecular diagnostic assays, and correlation between genotype and non-clinical traits [23]. Similarly, the Genome-Phenome Analysis Platform (GPAP) facilitates data sharing as it now opens for submissions of projects from all users, and not only from RD-Connect partners [24]. One of their main objectives is to help the contributed projects to quickly make their data available to the broader community of rare disease researchers [25].
**3.2 Validation of Concepts**
  Most of the identified concept libraries from the literature have described their validation methods either in their published studies, or in their websites, or in both of them. For example, in the CALIBER platform, EHR-derived phenotypes have been extensively validated using six different approaches: cross-EHR source concordance, case note review, consistency of risk factor-disease association from non-EHR studies, consistency with prior prognosis research, consistency of genetic association, and external populations. The builder of the platform acknowledged that the case study would inform which validation(s) are most important. For example, phenotyping algorithms developed for disease epidemiology (e.g., screening or disease surveillance) might be designed for higher sensitivity whereas those used in genetic association studies might be designed to maximize positive predictive value (PPV) [21].
  The developers of the PheKB have developed the Data Dictionary / Data Validation Tool, which validates covariate data descriptions and related data and is a tool embedded for registered users. The user uploads to the phenotype-related page, and the tool verifies the data dictionary file for compliance with standards and best practises. A specified set of rules defines differences from the standard or guidelines for best practises [18].
  The Concept Dictionary and Glossary was built at MCHP to assist researchers to carry out methodologically comprehensive research using consistent, validated algorithms [20]. According to their builders, concepts are written using original ideas and methods developed for MCHP reports, then reviewed and shaped according to common standards by the repository analyst [26]. Similarly, the data submitted including individual genomic and phenotype data, analytical results, general study information, are subject to quality checks by Genotypes and Phenotypes Database (dbGaP) staff before the Genotypes and Phenotypes Database (dbGaP) information is released publicly [15].
**3.3 Reusing of Concepts**
  All of the seven concept libraries allow reusing of stored data, clinical code lists and algorithms, however each concept library has established certain terms and searching features for users. For example, any user can download code lists from the ClinicalCodes.org repository. Once deposited, code lists will be freely available, with no login needed to download the codes. In addition, an open-source R package has been developed to automate the downloading of code lists from the online repository [9]. Also, the CALIBER platform allows reuse of existing lists of codes by researchers. Users can access phenotyping algorithms defining over 90 diseases and metadata. CALIBER has CALIBERcodelists package [27], which enables users to search for code lists by synonym or code stub, allows users to combine search terms using Boolean operators, and supports regular expressions for more advanced search queries. In addition, it allows downloading of the list of codes and some basic metadata for example, the name and version of the code list as a csv file [21].
  Algorithms and multiple implementation results can be publicly viewed in the PheKB website when authors designate it as “final”. By using metadata, users can search an algorithm based on inclusion or exclusion of data elements classes, such as diagnosis, author, or keyword. Currently, there are 414 users of PheKB from 52 different institutions. The median of used algorithms per institution is four. As of March 2020, PheKB include 30 public algorithms with 66 executions and 62 non-final algorithms with 83 executions in different stages of development [18].
  PhenoScanner V2 is a searchable library of findings from large-scale genetic association studies which are publicly accessible. The database now includes more than 350 million association results and more than 10 million original results genetic variations [22]. The developers of the PhenoScanner V2 specified the terms of use in their website: 1) users should cite both their papers in any publication or presentation 2) users should cite the original paper where the results were obtained, including the references for the linkage disequilibrium statistics and variant & phenotype mappings where used and 3) users should comply with any other terms relating to the data [28].
  The Concept Dictionary and Glossary at MCHP describes more than 300 research concepts developed at MCHP for the analysis of data contained in the data warehouse hosted at MCHP [29]. Over time, traffic on the MCHP website has increased. Their analysis software, Deep Log Analyser, recorded more than two million visits in 2018. In addition, the Charlson Comorbidity Index (CCI) including a glossary of terms and concepts has been ranked as the most widely viewed definition in the MCHP Concept Dictionary for many years. Similarly, the Elixhauser Comorbidity Index concept has regularly appeared among the top five most viewed concepts; whereas measures of comorbidity have often been among the most viewed concepts [26].
  The Genotypes and Phenotypes Database (dbGaP) provides free access to publicly available information on completed research and studies-related documents. However, individual level data is open to scientists around the globe via controlled access application. This platform allows researchers and clinicians to quickly interpret and compare DNA sequencing data with clinical knowledge, including those who do not have training in bioinformatics. Information in the Genotypes and Phenotypes Database (dbGaP) is organized as a hierarchical structure and includes the accessioned objects, phenotypes (as variables and datasets), various molecular assay data, analyses and documents. The Genotypes and Phenotypes Database (dbGaP) enables both simple as well as advance searches [15].
4. **Limitations**
  Some of the developers of the concept libraries mentioned some of their limitations as described below:
  The ClinicalCodes repository does not offer methods for downloading code-lists and article metadata in machine readable form according to their developers, and is lacking search features needed to facilitate queries such as searching and downloading of codes by disease group, keyword and or code group, all of which are planned to be added in the future. Also, they stated that it does not have a protocol for enforcing quoting of the downloaded code lists. Therefore, it would be difficult to connect code lists from earlier studies [9].
  The developers of CALIBER mentioned that there are some fairly complete measures in CALIBER’s data, for instance, 82.6 percent of people with at least one measurement of BMI using the concepts in the library. But some measurements are less comprehensive, for example, only 44.9 percent have at least one total measure of cholesterol when using the library concepts. They also mentioned that different records in CALIBER can represent the same event or subsequent events at similar points in time. For example, fatal myocardial infarction can be reported in up to four diverse sources that vary in their specificity in diagnosis and precision in timing [21].
  The developers of PheKB stated that some algorithms cannot work at a given site as well as at another, and validation is the only way to distinguish poorly performing algorithms. They also mentioned that PheKB does not have programmatic interfaces with some of the networks needed to enable fast exchange of executable phenotyping algorithms [18].
  The developers of dbGaP declared that the National Institutes of Health (NIH) policies, such as restricting the context of available data only to researchers who consented to general research use, leads to limiting the related phenotype data to specific demographic and disease status information, reducing the data download capabilities of the browser, and showing a browser watermark that images must not be recorded or published [15] [31].

**Table 2: Some of the characteristics of the seven concepts libraries:** 

Concept Libraries	Access to the underlying data sources	Sharing/Uploading of Concepts	Reusing/Downloading of Concepts
1.General libraries	

ClinicalCodes.org	In the top menu tab 'Browse published studies,' the user may choose a published study from the list. It then shows all the code lists associated with that study.	"Users must register with ClinicalCodes.org (in the menu bar login/signup) and choose 'upload codes.' First, they need to add some metadata of the published study and then they can upload several codes lists as delimited text files into that study.Metadata and links to studies code lists could be shared in a machine-readable form using the available open-source R package (rClinicalCodes).	Code lists are released on ClinicalCodes.org using a Creative Commons Attribution 3.0 Unported License (CC BY 3.0), and a file containing all codes associated with a study can be downloaded and used freely by any user.Downloading individual code lists is a single-click process that does not involve logging in or supplying user information.Users can choose to explore and download some or all of the code lists as csv files.
CALIBER data portal	Access to CPRD linked data on the CALIBER portal complies with the governance policies for data access by the CPRD.Researchers should first sign agreements with UCL to access CPRD data. Non-UCL partners must apply for CPRD to become a CPRD-approved partner and sign a UCL-approved sub-license agreement.	If a project proposal for a researcher has been accepted, a registration with the UCL Identifiable Data Handling Service (IDHS) will be arranged in order to create a new share for the project on the safe haven.The data facilitator at CALIBER will direct researchers through the entire process.	All definitions of research variables that use CALIBER data sources are publicly available and can be accessed in human and machine-readable formats.CALIBER codelists package enable users to search for code lists by synonym or code stub, combine search terms using Boolean operators, and download the list of codes and some basic metadata as a csv file.
The MCHP Concept Dictionary and Glossary	The Data Access Process (DAP) of the Manitoba Centre for Health Policy (MCHP) are the processes that a researcher has to complete to access the data and conduct research using the Manitoba Population Research Data Repository.	Researchers can share their work, such as creating new concepts or updating existing concepts.The concept development guidelines are defined in the Concept Development Template. Concept Development Template.	**Concept Dictionary:** More than 300 research concepts developed at MCHP are publicly accessible. **Glossary:** Terms of documentations widely used in population-based research are freely available. Browse/Search the Concept Dictionary and Glossary
Phenotype Knowledgebase (PheKB)	Private Phenotypes with "In Development" status, phases of "Testing," or "Validation" are not publicly accessible, which can only be accessed if the user is logged in and the phenotype was shared with the user via one of the two collaborative groups: Owner Group Phenotypes or View Group Phenotypes.	Researchers can upload:Related documents and their phenotypes along with multidimensional metadata labels. Documents including detail descriptions of the computable algorithms, such as types of used data, logic of execution, definitions of data, and flow charts.	Algorithms and multiple implementation results can be publicly viewed in the PheKB website when author designated it as “final”.By using metadata, users can search an algorithm based on inclusion or exclusion of data elements classes, such as diagnosis, author, or keyword.

2. Specialized libraries	

Genome-Phenome Analysis Platform (GPAP)	Only approved users who have completed the registration and verification process can access the data stored on the GPAP. Users must be affiliated with a recognized academic institution as accredited clinicians/researchers and must demonstrate their approval of the RD-Connect Code of Conduct by signing theAdherence Agreement.	Data sharing is open for project submissions from all users, not only from partners of RD-Connect, but they have to register first in the GPAP website.The GPAP enables clinicians and researchers who upload patient datasets to analyse their own data.	Registered users are allowed to access and search data sets provided by other researchers on similar patients.Registered users can match make, find second families, and find patient populations for validation studies.
The PhenoScanner V2	Some of the datasets are available for download including: dbSNP 147 with variant annotation from VEP, Linkage disequilibrium statistics from 1000 Genomes and a subset of the processed GWAS datasets, but users should first contact phenoscanner@gmail.com to get an approval.	Users can input one genetic variant, gene, genomic region or trait in the home page text box (www.phenoscanne.medschl.cam.ac.uk) or upload as a tab-delimited text file up to 100 genetic variants, 10 genes or 10 genomic regions.	"Users can use the archived findings from large-scale genetic association studies which are publicly accessible.Information provided by project members are unrestrictedly accessible.
Genotypes and Phenotypes Database (dbGaP)	Free access to information on completed studies are open to the public.Individual level data is accessible to scientists around the world through controlled application of access.	NIH-funded researchers can share their produced data. However, studies that are not sponsored by the NIH, individual NIH Institutes and Centres (IC) make judgments about whether non-NIH sponsored data should be accepted.	Open-access data can be accessed online or downloaded without prior authorization or permission from dbGaP.Individual level data download requests are handled through the dbGaP Authorized Access System (dbGaPAA), a web portal that manages request submissions, and enables safe high-speed large data download for authorized users.

## Discussion

### Statement of main findings

Globally, the development and use of concept libraries is important for reusable health studies. A number of data linkage centres around the world have developed different concept libraries to facilitate repeatable research. This paper examined seven concept libraries, and variations in their definitions, names, types, functions, coding systems, and data access restrictions. One of our findings is that these concept libraries have developed independently and so are duplicating work but in slightly different ways.

For wide use of concept libraries, collaboration across data linkage centres is needed to develop common standards that govern and guide these emerging libraries. For example, they should agree on a relatively standard definition/name for concept libraries to enable users to locate them and then use them easily. In addition, builders of concept libraries should cooperate with each other to increase awareness about their existence and their various functions. What one concept library might do that others do not do (e.g., provide SNOMED or BNF code lists or provide definitions for demographic variables such as smoking, BMI algorithms that other concept libraries do not do). Raising awareness of the features in the different libraries could increase the contributions of users to these libraries and accelerate their wide adoptions.

For a comprehensive adoption of concept libraries, their various functions, such as enabling users to share, validate, and reuse concepts (e.g., code lists), and their search features should be assessed by developers, funders, users, and experts to ensure that they meet the needs of various users including researchers, clinicians and data analysts. Since there are two different types of concept libraries 1) general libraries that hold phenotypes of multiple specialties 2) specialised libraries that manage only certain specificity such as rare diseases, users' preferences for the type of concept library types needs to be evaluated (e.g., through interviews, focus group, and surveys) before developing new concept libraries.

### Strengths and limitations

This is the first study, to our knowledge, aimed at identifying existing concept libraries, exploring their various characteristics, and examining the current practises in this evolving field. Finding studies about a concept library for electronic health data phenotypes in the literature was challenging as there are a limited number of related studies. Another challenge was the lack of a standard name or definition that describes this kind of library. Therefore, a range of keywords were needed to make queries as efficient as possible. This paper studied only publicly existing dictionaries / libraries, and did not examine non-publicly accessible concept libraries which have a restricted accessibility through the network of the hosting organizations / institutes.

## Conclusion

The seven libraries identified have been developed independently and appear to replicate in different ways similar concepts. Collaboration between similar libraries would greatly facilitate the use of these libraries for others. The process of building code lists takes time and effort. Access to existing code lists increases consistency and accuracy of definitions across studies. Concept library developers should collaborate with each other to raise awareness of their existence and of their various functions, which could increase users’ contributions to those libraries and promote their wide-ranging adoption.

## Acknowledgments

Kuwait Cultural Office in London, HDRUK and the National Centre for Population Health and Wellbeing supported this research. 

## Ethics statement

Ethical approval to conduct the research was approved was provided by the Research Ethics Sub-Committee, Swansea University. 

## Abbreviations

CALIBER	Clinical disease research using Linked Bespoke studies and Electronic health Records
CPRD	Clinical Practice Research Datalink
EHR	Electronic health records
GPAP	Genome-Phenome Analysis Platform
HES	Hospital Episode Statistics
ICD	International Classification of Disease
MCHP	Manitoba Centre for Health Policy
NLP	Natural Language Processing
PEDW	Patient Episode Database for Wales
PPV	Positive Predictive Value
PheKB	Phenotype knowledgebase
SAIL	Secured Anonymised Information Linkage
SMR	Scottish Morbidity Records
THIN	The Health Improvement Network
UCL	University College London
dbGaP	Genotypes and Phenotypes Database
